# Metagenomic and metatranscriptomic profiling of *Lactobacillus casei* Zhang in the human gut

**DOI:** 10.1038/s41522-021-00227-2

**Published:** 2021-07-01

**Authors:** Jicheng Wang, Jiachao Zhang, Wenjun Liu, Heping Zhang, Zhihong Sun

**Affiliations:** 1 Key Laboratory of Dairy Biotechnology and Engineering, Ministry of Education P. R. C., Key Laboratory of Dairy Products Processing, Ministry of Agriculture and Rural Affairs China, Inner Mongolia Agricultural University, Hohhot, China; 2 School of Food Science and Engineering, Key Laboratory of Food Nutrition and Functional Food of Hainan Province, Hainan University, Haikou, Hainan China

**Keywords:** Molecular evolution, Microbiome

## Abstract

Little is known about the replication and dynamic transcription of probiotics during their “passenger” journey in the human GI tract, which has therefore limited the understanding of their probiotic mechanisms. Here, metagenomic and metatranscriptomic sequencing was used to expose the in vivo expression patterns of the probiotic *Lactobacillus casei* Zhang (LcZ), which was compared with its in vitro growth transcriptomes, as well as the dynamics of the indigenous microbiome response to probiotic consumption. Extraction of the strain-specific reads revealed that replication and transcripts from the ingested LcZ were increased, while those from the resident *L. casei* strains remained unchanged. Mapping of all sequencing reads to LcZ genome showed that gene expression in vitro and in vivo differed dramatically. Approximately 39% of mRNAs and 45% of sRNAs of LcZ well-expressed were repressed after ingestion into human gut. The expression of ABC transporter genes and amino acid metabolism genes was induced at day 14 of ingestion, and genes for sugar and SCFA metabolism were activated at day 28 of ingestion. Expression of rli28c sRNA with peaked expression during the in vitro stationary phase was also activated in the human gut; this sRNA repressed LcZ growth and lactic acid production in vitro. However, the response of the human gut microbiome to LcZ was limited and heterogeneous. These findings implicate the ingested probiotic has to change its transcription patterns to survive and adapt in the human gut, and the time-dependent activation patterns indicate highly dynamic cross-talk between the probiotic and human gut microbes.

## Introduction

Probiotics are defined as live microorganisms that confer health benefits to the host when present in adequate amounts^1^, which are one of the most commonly consumed dietary supplements. The concept of probiotic consumption, referring to dietary live bacteria supplementation, has sustained the continuous growth of the market^2,3^. Probiotic bacteria have been extensively studied for their wide utilization in dairy foods^4^ and prophylaxis and control of a number of diseases^5–7^, primarily focusing on their fate, activity, and impact on the human gut microbiota^8–10^, However, probiotic strains are generally part of our transient microbiome, which competes for ecological niches with indigenous microbiota and commonly includes bacterial strains in the *Lactobacillus* and *Bifidobacterium* genera^8,11^. It is still unclear whether probiotic microorganisms can replicate during their “passenger” journey in the human GI tract under the effects of host-derived selection pressures, which has therefore limited the understanding of their probiotic mechanisms.

Probiotics have been reported to benefit human health in different ways. The capability of probiotics to rapidly metabolize certain carbohydrates to lactic acid, acetic acid, or propionic acid may influence dietary carbohydrate degradation and alter the metabolic output, for example, the production of short-chain fatty acids (SCFAs) such as butyrate^8,12,13^. Many probiotics can establish colonization resistance and competitive exclusion of pathogens^14^. Some probiotics are reported to stimulate the human immune response^1,15–17^. However, the molecular mechanisms behind these functions remain largely elusive. Interestingly, a meta-transcriptomic study revealed elevated expression of genes encoding enzymes for carbohydrate utilization in the mouse gut microbiota^18^. The contribution of probiotic bacteria and host-microbiota in expressing these functional genes requires further exploration.

Knowledge of probiotic gene expression in the complicated gut microbe community using traditional methods has been limited both by its low abundance and by the presence of closely related species. Meta-genomic and meta-transcriptomic approaches have recently emerged as a powerful way to study the impact of pathogens and diet on modulating the composition of the human gut microbiota^19–21^. A recent study applying these technologies showed that the transient colonization of probiotic bacteria in the human gut mucosa was highly individualized^22^. Another study also mapped meta-transcriptomic reads obtained from fecal samples from elderly volunteers onto the probiotic *L. rhamnosus* GG, showing high expression of LGG at 28 days of ingestion in some elders^23^. These reports prompted us to explore the possibility of using meta-transcriptomic reads to study the dynamics of probiotic transcription in the human gut. However, our knowledge of how the transcriptomes of global intestinal microbes in the human GI tract respond to probiotic consumption is limited.

Here, we took advantage of the high-throughput metagenomic and metatranscriptomic sequencing reads obtained from fecal samples of healthy young volunteers before and during probiotic ingestion and extracted strain-specific reads to explore the in vivo colonization, replication, and transcription of ingested *Lactobacillus casei* Zhang (LcZ), a koumiss-derived probiotic lactic acid bacterium, has been demonstrated to improve gut health^24,25^. At the genomic scale, the LcZ consists of a 2,861,848 bp circular chromosome and a 36 kb plasmid with 2, 804 and 44 predicted coding sequences (CDSs), respectively^26^. In our previous study, we found that LcZ strains had the more abundant PTSs and 2CRSs among completely sequenced LAB, and some PTS families in LcZ such as EII^Gat^, EII^Man^ and EII^Asc^ expanded particularly, which enhanced its capacity to use various carbohydrates with higher efficiency and make it more adaptable to the complex gut environments. Comparative genome analysis also revealed some genetic basis for its probiotic properties, such as adherence to the epithelial cell surface and EPSs biosynthesis, which would certainly promote its colonization in the host gut^26^. Over the last 15 years, the LcZ was widely used in clinical research (or animal model) and exhibited excellent health-promoting properties compared to other *L. casei* isolates. For example, the LcZ could alleviate shrimp tropomyosin-induced food allergy by switching antibody isotypes through the NF-κB-dependent immune tolerance^27^; LcZ maintained the intestinal microbiome homeostasis of the sailors and prevented sailor anxiety during a long sea voyage^28^; the fermented milk containing LcZ alleviated constipation symptoms through regulation of intestinal microbiota, inflammation, and metabolic pathways^29^; LcZ prevents jejunal epithelial damage to early-weaned piglets Induced by *Escherichia coli* K88 via regulation of intestinal mucosal integrity, tight junction proteins and immune factor expression^30^. Accordingly, we further compared the transcriptomic profile of LcZ in vivo and in vitro samples and mainly focused on differences in mRNA and sRNA expression. Finally, we described the taxonomic and functional dynamics of the indigenous gut microbiota response to LcZ consumption. These novel findings underline the high regulation of the probiotic genome after ingestion into the human GI tract.

## Results

### The colonization and replication of *L. casei* Zhang (LcZ) strains in the human gut

**Fig. 1:**
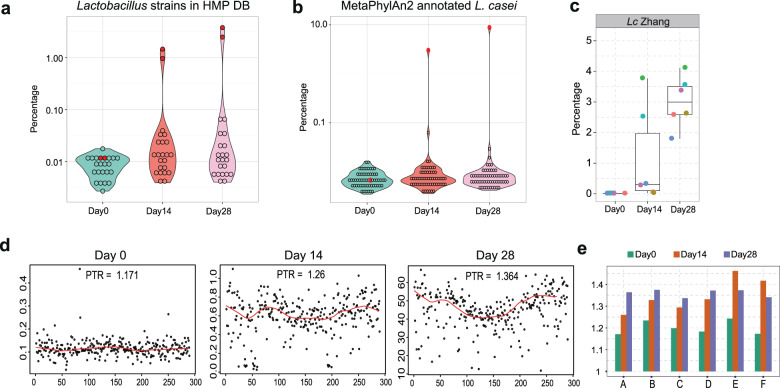
The increasing replication of *L. casei* Zhang in the human gut. **a** Violin plot of total TPM for 23 *Lactobacillus* strains in HMP database for meta-genomic reads. Each dot represents the sum fraction of six samples in one time-point. The two red dots represent the two *L. casei* strains in HMP database. **b** Violin plot of MetaPhylAn2 mapping of meta-genomic reads. The red dot represented *L. casei*. **c** Box plot of the mapping percentages of meta-genomic reads to the *L. casei* Zhang. **d** Example of genome coverage and PTR values for A volunteer. *X*-axis represents the genomic location by 10 kb, and *Y*-axis represents the genomic coverage. The black points represent the coverage in each location, and the red lines represent the fitting curve for the points. **e** Barplot of the PTR values in each stage and each volunteer.

To quantify the colonization and replication of LcZ in the human gut, we first analyzed the mapping dynamics of 23 *Lactobacillus* strains collected in the HMP database (Supplementary Table 1), including two other *L. casei* strains. These two strains show symmetric sequence identities of 91.99% and 85.19% with LcZ. Prior to ingestion, the gut meta-genomic DNA mapped to all *Lactobacillus* strains constituted 0.11–0.30% of the total mapped DNA, and all strains were presented at similar fractions (Fig. 1a). Upon ingestion, the fractions of DNA mapped to two *L. casei* strains increased up to two orders of magnitude, while those to the other *Lactobacillus* strains only changed slightly. Then, we further analyzed the meta-genomic reads using the MetaPhlAn2-based *Lactobacillus* strain database (Supplementary Table 2), which only recovers the reads uniquely mapped to each strain and therefore effectively excludes the noise of multiple mappings caused by sequence conservation and strain similarity. The MetaPhlAn2 results revealed a much more pronounced and absolute increase in the mapped fraction of this *L. casei* strain. The total increase reached up to 10% of the gut microbiota (Fig. 1b). To distinguish the contributions of the ingested LcZ and resident *L. casei* strains to the meta-genomic reads, we mapped the reads to the genomes of LcZ. The uniquely mapped reads were extracted for statistical analysis in all the meta-genomic samples. An exclusive increase in the reads uniquely mapped to the LcZ genome was evident (Fig. 1c).

The large population of ingested LcZ in the human gut indicated probiotic bacterial replication. The above results set the stage for us to further analyse the replication of the ingested probiotic replicate during the duration of their “passenger” life in the human gut by using the recently published peak-to-through (PTR) approach^23^. The higher replication rate results in larger variation and PTR value. We implemented the approach and calculated PTRs of LcZ in each sample based on the meta-genomic reads mapped onto the LcZ genome (Supplementary Table 3). Given that the increased *L. casei* mapping was due to the ingested LcZ but not the resident *L. casei* strains, reads mapped onto *L*. LcZ could reflect the resident strains at day 0 and ingested *L. casei* at day 14 and day 28. The PTR-calculated position of the replication terminus based on meta-genomic reads overlapped well with the terminus calculated (Fig. 1d and Supplementary Fig. 1). Our calculation yielded a PTR of ~1.2 for the resident *Lactobacillus* strains in all individuals (day 0) (Fig. 1e). The PTRs increased to 1.25–1.45 at 14 days of ingestion and stabilized at ~1.35 at 28 days of ingestion, indicating that the ingested LcZ actively replicates in vivo at a higher replication rate than those of the resident *Lactobacillus* strains.

### Comparison of the genome expression patterns of LcZ in vitro and in the human gut

**Fig. 2:**
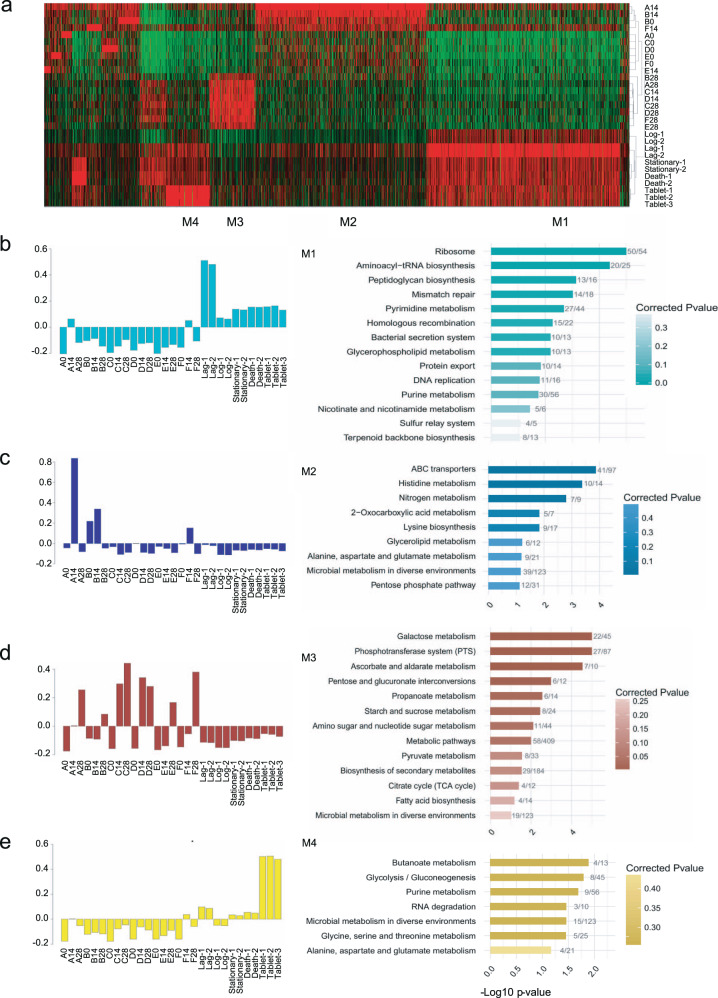
Transcriptional dynamics of the ingested *L. casei* Zhang in the human gut. **a** Heatmap representation of differentially expressed genes mapped onto the *L. casei* Zhang genome, ranked by the co-expression modules. **b**–**e** Bar plots of eigengene value and KEGG pathway enrichment of corresponding genes in module 1 (**b**), module 2 (**c**), module 3 (**d**), and module 4 (**e**).

We next compared the transcriptome of LcZ in vitro and in the human gut. We were aware that the in vivo expression of LcZ was mixed with a fraction of resident *L. casei*. However, the relative abundance of resident *L. casei* was very limited. Therefore, the contribution of the gene expression of *L. casei* in vivo was mainly sourced from LcZ. Differentially expressed genes were obtained within the in vivo or in vitro groups, as well as between the in vivo and in vitro groups, which were subjected to WGCNA network analysis. Almost all *L. casei* genes (97.22%; 2871/2953) could be detected in transcriptional regulation during in vitro and in vivo growth of the probiotic (Fig. 2a). The M1 module contained 948 genes, representing 32.1% of all *L. casei* genes, which were expressed very well when grown in vitro but strongly repressed when grown in the human gut. These genes were expressed at relatively higher levels in two day-14 samples (individuals A and F), which could reflect transcripts from the transiently passed LcZ after being ingested. M1 module genes were enriched in KEGG pathways for translation and replication (Fig. 2b).

Expression of *L. casei* genes in M2 modules (839) was induced at day 14 in three samples. These genes were strongly enriched in ABC transporters and metabolism pathways of multiple amino acids (Fig. 2c), suggesting the possible presence of a transition stage, during which the ingested LcZ has to alter its uptake function to adapt to the human gut environment. Genes in M2 modules highly overlapped with the genes in the turquoise module. At day 28, the late stage of ingestion, the expression of a cluster of genes (226) was specifically increased (M3 module). These genes were involved in the biosynthesis and/or metabolism of well-known probiotic molecules, including galactose, carbohydrate utilization, and metabolism of propanoate, the key member of SCFA (Fig. 2d). We found that LcZ genes for ascorbate and aldarate metabolism were globally upregulated, suggesting a novel class of probiotic molecules. Genes in the M4 module (215) were mostly expressed in the tablet form of LcZ, and their level in the human gut was increased at the late stage of ingestion (Fig. 2e). M4 genes were most strongly enriched in the metabolism of butanoate, another key member of SCFA synthesis.

### Dynamic expression of sRNA genes of LcZ in vitro and in vivo

**Fig. 3:**
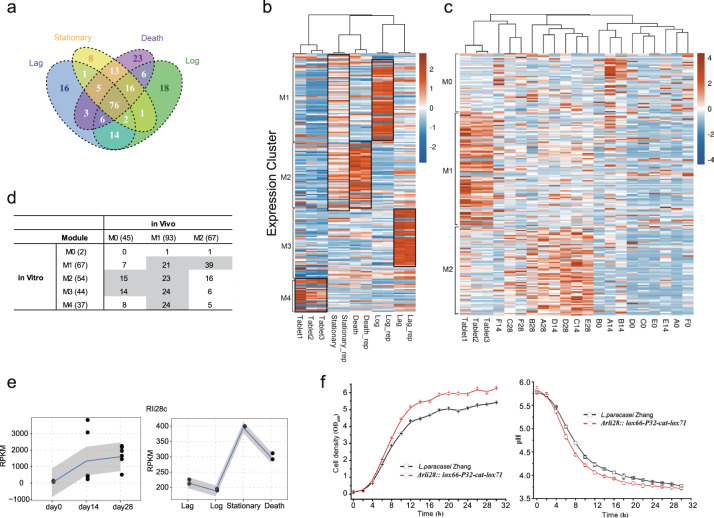
Expression profile of sRNAs and the function of rli28c sRNA. **a** Venn diagram showed the sRNA detection overlap among the four growth stages in vitro. **b** Heatmap presentation of the expression pattern for the in vitro and tablets samples by WGCNA clustering. Black rectangle represents the highly expressed sRNAs in the corresponding samples. **c** The same with (**b**) but for the in vivo and tablet samples. **d** The overlapped sRNAs numbers for major modules classified by WGCNA for in vitro and in vivo shown in (**b**) and (**c**). **e** The expression level line plot of RPKM values for rli28c sRNAs in in vivo and in vitro samples, respectively. **f** The cell density (left) and pH value of the growth medium (right) plot by time with (red) and without (black) the rli28c sRNA knockout. Three biological replicates were used in this experiment.

Given the regulatory function of bacterial sRNAs, we then studied the possible contribution of sRNA to the highly dynamic transcriptome of LcZ. A total of 208 candidate sRNAs were identified from the in vitro samples. Among these candidate sRNAs, 76 were identified from all four stages, and 143 were identified from at least two growth stages (Fig. 3a). A heat map of the expression patterns of all sRNAs in the in vitro growing states showed that although most sRNAs were expressed under multiple growth conditions, stage-specific expression of sRNAs was prevalent for LcZ (Fig. 3b). Lag phase, log phase, death phase, and tablet phase sRNA clusters were highly specific (Fig. 3b). Interestingly, the stationary phase did not contain specific sRNA, and bacteria expressed sRNA specific for the log and death phases at relatively high levels (Fig. 3b). When *L. casei* was expressed in the human gut, the expression of sRNAs was clearly separated into two clusters. The M1 sRNAs decreased their expression after ingestion, while the M2 sRNAs increased their expression in comparison with the sRNA expression in the tablets (Fig. 3c). The in vivo M1 sRNAs contained sRNAs specifically expressed at each of the four in vitro growth stages at an unbiased frequency, while M2 sRNAs were mainly those of the in vitro log phase sRNAs (Fig. 3d). This observation suggested that the in vivo growth state of LcZ might resemble the in vitro log phase.

Rli28 is a small RNA (sRNA) that is detected in *Listeria monocytogenes* grown in the stationary phase and in the intestinal lumen of infected mice and proposed to be involved in bacterial virulence^31^. We identified five copies of rli28 expressed from the genome of LcZ, ranging from 210 to 492 bp and located at two separated loci (Supplementary Table 4). The levels of LcZ rli28 genes varied greatly between the strain grown in vitro and in vivo (Fig. 3e, Supplementary Table 5). The in vitro expression patterns of these rli28 genes of LcZ differed significantly, with one peaking at the log phase (rli28e), two peaking at the stationary phase (rli28c and rli28d), and two peakings at both the stationary and death phases (rli28a and rli28b). The expression of four rli28 genes in the human gut constantly increased with the ingestion time, while the rli28a gene expression was decreased at day 28.

Rli28c peaked at the stationary phase and was chosen for further functional analysis (Fig. 3f). After rli28c was knocked out using the Cre/LoxP cassette, the in vitro growth of the mutant LcZ was enhanced compared to the wild-type (Fig. 3f). In addition, the growth medium pH of the mutant LcZ was lower than that of the wild-type, consistent with an enhanced release of lactic acid. Taken together, these results suggested that the stationary phase rli28c may repress the growth and production of lactic acid by LcZ. To confirm the above results, we also measured directly the presence of lactate and SCFAs in the parental strain and mutant. We uncovered that the absence of rli28c was beneficial for LCZ to produce more lactic acid, acetic acid, propionic acid, and butyric acid (Supplementary Fig. 2).

### The response of human gut microbiota to LcZ was limited and heterogeneous

**Fig. 4:**
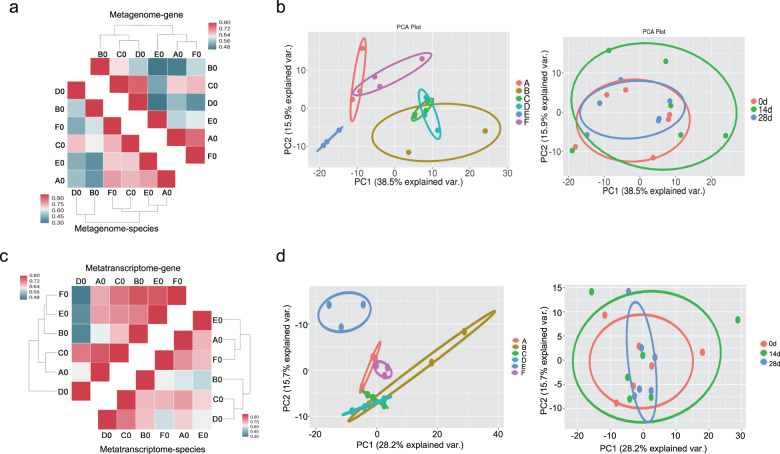
The response of human gut microbiome to *L. casei* Zhang was limited and heterogeneous. **a** Hierarchical clustering heatmap of sample classification by Pearson correlation coefficient of genes (upper right) and species (lower left) abundance prior to *L. casei* Zhang ingestion. **b** PCA analysis of meta-genomic species abundance in all 18 samples by individual classification (left) and temporal classification (right). **c** Hierarchical clustering heatmap of sample classification by Pearson correlation coefficients of species (lower right) and gene (upper left) abundance prior to *L. casei* Zhang ingestion. **d** PCA analysis of meta-transcriptome species abundance in all 18 samples by individual classification (left) and by temporal classification (right).

After obtaining the meta-genomic data, we studied the impact of LcZ ingestion on the gut microbial community by analyzing the meta-genomic reads. Prior to LcZ ingestion, a large inter-individual difference in gut microbiota at the gene and species levels was observed (Fig. 4a). Upon LcZ ingestion, both sample correlation analysis and PCA analysis showed that the probiotic-induced microbiota composition change was generally much smaller than the inter-individual difference (Fig. 4b, Supplementary Fig. 3). The effect of the ingested LcZ on the major bacterial populations at different levels reflected by MetaPhlAn2 analysis was also inconsistent among individuals (Supplementary Fig. 4). Interestingly, *Lactobacillales* and *Lactobacillus* at the order and genus levels were both consistently increased in some of the 14-day and all 28-day samples among all six individuals. Then, we further studied how LcZ affected the transcription/function of our volunteers’ gut microbiota by analyzing the meta-transcriptomic data obtained from the same fecal samples as those of the meta-genomic data. Expression correlation analysis showed a smaller inter-individual variation among metatranscriptomes than the metagenomes (Fig. 4c). The probiotic-induced change in metatranscriptomes was similar and even smaller than that of metagenomes at both the species and contig levels (Fig. 4d, Supplementary Fig. 5), confirming the lack of a global transcriptional response. Although we observed slight fluctuations in the composition of metagenome and metatranscriptome from 0 to 14 days and 14–28 days, no significant difference was found at different time points (Fig. 4b, d, Adonis test *P* > 0.05). We hold the view that the individual characteristics of different hosts are much greater than the effects of probiotic intake on intestinal microbiota, and similar studies have been reported before^24^. Notably, the metagenomes composition of individuals B and D at days 0 and 28 were more similar to those at days 0 and 14, whereas the metatranscriptome in the gut was the opposite. Similarly, the intestinal metagenome composition of individual C at days 0 and 28 was less similar than that between days 0 and 14, and the metatranscriptome was the opposite. The uniform results were only observed in individuals A, E, and F (50% individuals). Therefore, in addition to investigating the gut metagenomic composition, metatranscriptome profiles of gut microbiota should also be evaluated.

## Discussion

Currently, although the transcription pattern of *Lactobacillus* in vitro and in the murine gut has been investigated^32,33^, however, exploring the fate of ingested probiotics thoroughly at the transcriptional level remains a challenge in the human gut. To the best of our knowledge, this study presented the first effort to profile the replication and transcription of mRNAs and sRNAs of probiotic and resident *Lactobacillus* in the human gut by extracting the transcripts of probiotic bacteria from meta-transcriptomic sequencing reads. Classical meta-genomic and meta-transcriptomic analysis show that the ingested probiotic bacteria do not alter the global intestinal microbiome to any appreciable level compared to the individual variations, consistent with previous results^34–38^. Probiotic microorganisms can generally survive well when they pass through stressful GI tract conditions in a few hours and stay in the colon for a few days^1,8,11^. Microbial cells that cannot survive the GI tract undergo cell lysis^8,39^. It is unclear what is going on at the transcriptome level when probiotics are ingested. Here, we have demonstrated that transcription of the ingested LcZ does not inherit the in vitro transcriptional pattern at all, which was similar to *Lactobacillus acidophilus*^40^. Moreover, transcriptional patterns at day 14 and day 28 differ significantly. These findings have an important implication regarding the fate of ingested bacteria and what we are detecting from the fecal samples. We are concerned that during the course of probiotic uptake, the majority of probiotics that we detected from fecal samples are dead bacteria after ingestion. However, the distinct transcription patterns between in vitro and in vivo, as well as between those after 14 and 28 days of probiotic uptake, strongly suggest that the detected probiotic transcriptomes reflect bacteria that have survived the GI tract. We do not exclude the possibility that the dead probiotic bacteria might still produce fragmented DNA signals. However, our results support the previous hypothesis of cell lysis for dead ingested bacteria^9,39^ and the dead probiotic is unlikely to yield RNA signals according to our reported transcription patterns. In conclusion, this study suggests that transcriptome analysis represents a more effective way to detect living bacteria in fecal samples.

ATP-binding cassette (ABC) transporters represent one of the largest classes of transporters using the power from ATP hydrolysis to drive the translocation of different substrates across cell membranes^41^. ABC transporters not only transport a large variety of nutrients into cells from the environment but also transport various cellular components away from the cells. For example, multidrug ABC transporters transport a wide range of drugs from cells^42^. In this study, we found that in three day 14 fecal samples and one day 0 fecal sample, genes encoding ABC reporters were globally activated in LcZ compared with their expression under in vitro growth conditions. As we have shown, upon LcZ ingestion, the increased *L. casei* mapping percentage is from the ingested LcZ. The increased expression of ABC transporters should therefore indicate that the LcZ-surviving GI tracts have changed their expression pattern, favouring the expression of ABC transporters. Activation of the expression of ABC transporters might enhance the ability of ingested probiotics to take up nutrients from the human gut environment. It may be related to genes encoding ribose ABC transporters and maltose ABC transporters component^43^. Notably, maltose ABC transporters have been functionally correlated with the metabolism of the prebiotics isomaltooligosaccharides, dextran, and maltotetraose^40^. Comparative genomic analysis showed that the number of phosphotransferase system (PTS)-related proteins in LcZ genome was abundant, including cellobiose, fructose, mannose, beta-glucoside, N-acetylgalactosamine, lactose, sucrose, trehalose, alpha-glucoside, sorbose, etc. Most of the above substrates are similar to other *Lactobacillus*, such as *Lactobacillus plantarum* WCFS1 (rhamnose, mannose, cellobiose, sucrose, maltose, lactose, trehalose, melibiose, N-acetyl-d-galactosamine, etc.)^44^, and *Lactobacillus johnsonii* (maltose, fructose, glucose, cellobiose, etc.)^32^, and particularly, *Lactobacillus reuteri* (xylose)^45^, *L. acidophilus* (isomaltose, isomaltulose, panose, and gentiobiose)^46^. Then, the similarity between the remaining day 14 individuals and day 28 individuals confirmed that the PTS was significantly enhanced, which proves that there may be a transition from ABC transporters to PTS transporters in LCZ genome at this stage. Our previous research showed that the related sugar metabolism genes may involve mannose, cellobiose, mannitol and sorbitol^43^. Coincidentally, mannose PTS was uncovered in long-gut-persistence strains (*L. johnsonii* strains NCC533 and ATCC 33200)^33^, which may be related to gut residence time. It is known that human gut microbes establish direct chemical interactions with the host^47^. It could be possible that the signals for the global activation of ABC transporters were produced by the gut microbial community, reflecting its early cross-talk with the ingested probiotic. On the other hand, activation of ABC transporters could also reflect how the ingested LcZ responds to living conditions in the human gut.

Moreover, we observed a clear shift of transcriptional patterns between day 14 and day 28 samples, in which the activated expression of the ABC transporter disappeared but activated expression of genes for galactose and sugar metabolism appeared. This shift indicates dynamic cross-talk between ingested LcZ and human gut microbiota. It is possible that the early cross-talk elicits a signal for activated expression of ABC reporters. However, as LcZ uptake continues, the interaction between the ingested LcZ and human gut microbes is established, and the signal for activation of ABC transporters of the ingested LcZ might be lost. Instead, signals for galactose and sugar metabolism are secreted, sensed and received by the ingested LcZ. The human gut microbiome develops with its host after birth, which modulates the host metabolic phenotype^47^. The host and microbiome establish metabolic axes resulting in combinatorial metabolism of substrates by the microbiome and host genome, which produce various metabolites such as bile acids, choline, and SCFAs that are essential for host health^48,49^. It is interesting to observe that transcriptional states of genes for galactose and sugar metabolism in the later stage of the ingestion of probiotic LcZ, as well as genes for the metabolism of one class of SCFAs (propanoate)^50,51^, were globally activated. Notably, starch is the main digestible carbohydrate component in the diet of mice, and starch and sucrose metabolism were activated at day 28, which reflecting the ability of LcZ to harvest carbon substrates from starch components, which may be targeted to α-1,4-/α-1,6-glucoside and β-glucoside^40^. The result is related to the apparent redundancy of the PTS encoded by LcZ chromosome, as it may provide benefits in the transport and use of large panel of carbon sources^26^. Additionally, the participation of external non-digestible oligosaccharides may also activate galactose and sugar genes (β-galactosidases) of *Lactobacillus*^52^, which could be attributed to the presence of these nondigestible oligosaccharides in the mouse chow^53^. These findings are consistent with the current knowledge that probiotic bacteria can contribute metabolites such as acetate, lactate and propanoate^8,54,55^ by metabolizing nondigestible oligosaccharides and starch. A number of reports have shown that *Lactobacillus* stains produce SCFAs^56,57^, and our previous research also found that the consumption of LcZ exhibited a prolonged elevation of SCFA and reduction of TBA^58^. Importantly, we found that the increase in propionic acid is dependent on the intake time and is much more pronounced after 3 weeks of intake than after 8 days, which agrees well with our observed time-dependent activation of genes for propanoate metabolism. It can also explain that short-term intervention of *Lactobacillus* may have limited effect on the production of SCFAs^59^. Overall, we need to examine the time effect of probiotics from the perspective of metatranscriptomic.

sRNAs represent a large class of novel regulatory molecules in bacteria^60,61^. The sRNAs in *Lactobacillus* have not been well characterized before. In this study, we identified 208 sRNAs in LcZ growing under four different growth stages in vitro, among which 76 overlapped. Almost all sRNAs display a stage-specific growth pattern, which agrees well with the regulatory roles of sRNAs^62,63^. After intake, we found that sRNAs highly expressed in the death and stationary phases were highly expressed in the human gut. By creating a lox knock-out LcZ, we have shown that one copy of *rli28* that is best expressed in the stationary phase inhibits LcZ growth in vitro. This suggests that sRNAs could regulate the bacterial growth rate. The unique transcription pattern of the probiotic bacteria in vivo might shape their characteristics of being transient passengers without much of an effect on the resident gut microbiota. These findings together underline the presence of dynamic cross-talk between the probiotic and human gut, including the microbial community, which ensures tightly regulated expression of the probiotic genome in vivo, which is worth further study in the future. Moreover, the developed methodology can be extended to study the in vivo expression of probiotics and pathogens.

## Methods

### Experimental design

The experiment was approved by the Ethics Committee of the Inner Mongolia Agricultural University (Hohhot, China). A written consent was obtained from every volunteer. Subjects were asked to orally intake four probiotic tablets consisting of a total of 10.6 Log_10_ CFU LcZ daily from Day 0 to 28. Stool samples were collected from the subjects on Days 0, 14 and 28 in sterile containers and were kept refrigerated. Gut microbiota was sampled by non-invasively fecal collection. Stool samples were taken in duplicate by coring out feces with inverted sterile 1 mL pipette tips. These tips were then deposited in 15 mL Falcon tubes, and RNAlater was added to the tubes for meta-transcriptomic sequencing. Samples were collected in 2 min at home and stored temporarily at −20 °C. Samples were then transported on ice to the laboratory within 2 h and then stored in −80 °C freezers immediately. In this study, we used LcZ as a model to study the in vivo transcription dynamics of ingested probiotics, as well as the response of indigenous microbiota to probiotic consumption. We collected metagenomic and meta-transcriptomic reads from the fecal samples taken from six healthy young volunteers (20–30 years old, three males and three females, labeled as A–E, Supplementary Table 6) in an open-label clinical trial. The fecal samples were taken on day 0 prior to the consumption and on day 14 and 28 after consumption. As controls, we obtained three replicated transcriptomes of LcZ in tablet form prior to the ingestion. In order to assess the growth condition of the probiotic in gut microbial community, we additionally sequenced the transcriptomes of LcZ cells growing in vitro at the lag, log, stationary and death phases.

### Gut microbe preparation

Gut microbes were released from about 5 g of each fecal sample by vigorous vortexing in DEPC waters in 15 mL Falcon tubes. The supernatant was immediately collected after natural sedimentation and followed by centrifugation at 8000×*g* for 3 min. The microbe pellets were collected for DNA and RNA preparation. Preparation of gut microbial total RNA and DNA libraries was described in the following methods.

### Genomic DNA extraction, library construction and sequencing

Genomic DNA was extracted from gut microbe samples using a protocol that lyses bacterial cells combining lysozyme, lyticase and 1% SDS. Gut microbe samples were sheared by DNA Shearing Instrument (30W%, 3sON, 9sOFF, 25 min). The size of the sheared DNAs was between 150 and 700 bp that was verified by 1.5% Agrose gel electrophoresis. The quantity of the sheared DNAs (after purification) was measured by Qubit 2.0 (Invitrogen). For each sample, 300 ng of the fragmented genomic DNA was used for pair-end library preparation. Following end repairing and A tailing, the DNAs were ligated to the double-stranded DNA adaptors. The ligated products were amplified with polymerase chain reaction (PCR). After that, PCR products corresponding to 300–500 bp were purified, quantified and stored at −80 °C until used for sequencing. For high-throughput sequencing, the libraries were prepared following the manufacturer’s instructions and applied to Illumina Hiseq 2000 system for 100 nt pair-end sequencing.

### PTR analysis for the growth rate of the bacterial population

In a bacterial population, every cell may be at a different stage of replication. The ratio between DNA copy number near the replication origin and that near the terminus, which we term peak-to-trough ratio (PTR), should reflect the growth rate of the bacterial population^23^. Before PTR calculation, we used the oriloc method to predict the replication origin of LcZ^64^. We aligned meta-genomic reads to the LcZ genome and selected the uniquely aligned reads as the final alignment result. After that, we binned the coverage of genomic segments into 10 kbp regions, and calculated the mean coverage (depth) in each bin. Then, we filtered the outliers that are more than 2 standard deviations from the mean. Finally, we smoothed the coverage of the resulting bins by the lowess method in R software. The PTR was the smoothed sequencing coverage of the representative strain at the predicted peak location divided by that at the predicted trough location.

### Fecal RNA extraction and sequencing

After obtaining all the samples, they were shipped to the ABLIfe Inc., Wuhan, China on dry ice, and then used to extract total RNAs. Extraction of total RNA was performed for all samples using Trizol Reagent (Invitrogen) according to the manufacturer’s instructions. Total RNAs were treated with RQ1 DNase (promega) to remove DNA. The quality and quantity of the purified RNA were determined by measuring the absorbance at 260 nm/280 nm (*A*260/*A*280, ~2.0) using smartspec plus (BioRad). RNA integrity was further verified by 1.5% Agrose gel electrophoresis. For each sample, 5 μg of total RNA was used for RNA-seq library preparation. Ribosomal RNAs were depleted with Ribo-Zero rRNA depletion kit (Epicentre, MRZB12424) before used for directional RNA-seq library preparation (gnomegen K02421-T). Purified mRNAs were iron fragmented at 95 °C followed by end repair and 5′ adaptor ligation. Then reverse transcription was performed with RT primer harboring 3′ adaptor sequence and randomized hexamer. The cDNAs were purified and PCR amplified. PCR products corresponding to 200–500 bps were purified, quantified and stored at −80 °C until used for sequencing.

### In vitro sample RNA extraction, library construction and sequencing

For the in vitro bacterial samples, we collected the samples in two different styles. As for the first style, we cultured the LcZ on the medium and collected two replicate samples from each of the four growth stages, lag, log, stationary, and death stage, respectively. For the second, we collected samples for the same probiotic tablets as the above, and three replicates were prepared. After sample collection, total RNAs were extracted from samples mentioned above by using Trizol Reagent (Invitrogen). Then we used Ribo-Zero rRNA (Epicentre, MRZB12424) removal kit to remove the rRNAs. After that, extracted RNA was amplified using custom barcoded primers and sequenced with paired-end 100 bp reads by illumina HiSeq2500 platform (ABLIfe Inc., Wuhan).

### Quality filtering and sequence statistics

After sequencing, raw reads would be first discarded if containing more than 2-N bases, then reads were processed by clipping adaptor, removing low-quality reads and bases from the end of each reads and discarding too short reads (<16nt) by FASTX-Toolkit (Version 0.0.13). The metagenomic, metatranscriptomic and the in vitro samples were filtered with the same method and parameters.

### Data validation by RT-qPCR

Genomic DNA and total RNA were extracted from the fecal samples of each volunteer. For metatranscriptomic mRNA detection, total RNAs were extracted from the same fecal samples of each volunteer for sequencing. To ensure there was no genome DNA contamination, RNA was treated with DNAse 1 (Takara) for 2 h, and then applied to PCR validation. The mRNA fragments of β-actin (human) obtained by in vitro Transcription (Transcript Aid T7 High Yield Transcription Kit, Thermo Scientific) was added into each RNA sample and applied to the reverse-transcribed by random hexamer primers using M-MLV reverse transcriptase (Promega). RT-qPCR was performed using ABI Prism 7300 Real-Time PCR System with standard procedure, and the relative expression level of genes was normalized by β-actin. The PCR primers were provided in Supplementary Table 7.

### HMP database retrieval and MetaPhlAn2 analysis

We chose HMP database (http://hmpdacc.org/) as a reference to do the structural and functional analysis. First, we downloaded the complete genome sequences and annotation of the human gut microbiome, which contains 358 publicly available human microbiome genomes generated from the National Institutes of Health (NIH) Human Microbiome Project and the European MetaHIT consortium. Besides, we added the LcZ genome (http://www.ncbi.nlm.nih.gov/) to the database to evaluate the influence of LcZ on the microbiome. We then aligned our metagenomic and metatranscriptomic data to the genomes with Bowtie2^65^, allowing no more than one mismatch. The MEGAHIT was used to generate assembled contigs^66^. After that, we calculated the reads number and RPKM (Reads Per Kilobase per Million mapped reads) value for each contig and gene in the database. We then obtained the abundance of different taxonomic levels from species to kingdom by adding relative contigs abundance together. To consistently estimate the functional composition of the samples, we annotated the genes from the HMP database using COG orthologous groups and KEGG pathways by blastx program with e-value 1e−5. We ensured that comparative analysis using these procedures was not biased by data-set origin, sample preparation, sequencing technology and quality filtering.

For meta-transcriptomic gene abundance, to study gene expression alteration changed by ingestion of LcZ, we compared the expression change between day 14 and day 0, day 28 and day 0 and day 28 and day 14. First, we got differentially expressed species (Wilcoxon rank-sum test) and extracted all genes abundance from these species, and then obtained the differentially expressed genes. We then used WGCNA^67^ method to classify the differentially expressed genes as modules based on their expression pattern. After classification, we used the annotation of KEGG to obtain the functional enrichment pathways by hypergeometric test.

For both meta-genomic and meta-transcriptomic reads, we have applied the MetaPhlAn2 and GraPhlAn software (54) to obtain the relative abundance of each species. Top abundant species of all samples were used to make a dendrogram heatmap via hierarchical clustering. After the calculation of species abundance, we got differentially expressed species to analyze the influence of LcZ on transcription variation.

### In vivo and in vitro samples co-expression analysis

To find the transcriptome difference of LcZ between in vivo and in vitro samples, we compared the gene expression difference among these samples by aligning the transcriptome reads to the LcZ genome. We used Bowtie2^65^ software to align reads to the LcZ genome allowing 1 seed mismatch. RPKM value for each gene was calculated for each sample. Then we compared the gene expression changes between each samples groups with each other by edgeR^68^ package. Samples in vivo of each point were compared with samples in vitro of each stage and type, and samples in vivo were compared with each other, samples in vitro were compared with each other. We then used WGCNA^67^ method to classify the differentially expressed genes as modules based on their expression pattern. After classification, we used the annotation of KEGG to obtain the functional enrichment pathways by hypergeometric test.

### Bacteria sRNA prediction and expression analysis

To have an exact prediction of LcZ sRNAs, we developed an algorithm to detect peaks from alignment results among intragenic, intergenic (between two adjacent genes) and antisense regions^69^. We used the RNA-seq data from four-stage bacterial strains cultured on the medium. We merged the mapping result file from the same stage and ran the computer program separately for the four stages. After prediction, we merged the sRNAs predicted from the four stages by genomic locations and got a final sRNA prediction result. We aligned the sRNA sequence to the Rfam database (version 12.0)^70^ to identify homologies from related bacteria by Blast method (e-value ≤ 1e−5). After sRNA prediction, we got the normalized expression level of each sRNA for each sample. We then used WGCNA^67^ method to classify the differentially expressed sRNAs as modules based on their expression pattern.

### sRNA knockout experiment

To validate the influence on bacteria by sRNAs, we selected sRNAs that expressed significantly and dynamically to do the knockout experiment. Rli28 and ratA (positive control) from the plasmid of LcZ were chosen. The target sequence of Rli28 is TTAATGCGATTAAAGCCACGGTAAAGGTACCGAAAGCCAGCATTAATTGTAAAGCGTCCGCAACGGACACTTAGGCTACTCCTTTCATTAGGATTTATGGGCTTTAGGGGTTTAACACCATAAGCACCACCTCCGATCGGAAATAGCCACCGCCTTAACTTCTCTACAAGCTTTAATTATACAGGAGCTTT, which locates on the plasmid from 30466 to 30656. The target sequence of ratA is TAATATAGACAGAAAAAGGGAAGCCCCGCTAGAACAGGACTTCCCATGCAAGCCGCTTCAAAGGCGGTGGCAGAAATTTAATAAACGATTTT, which locates on the plasmid from 28019 to 28110. The knockout experiment was performed according to one published protocol for gene deletions in *Lactobacillus*^71^, and the knockout efficiency of Rli28 was validated by RT-PCR. After knockout, we tested the cell density and pH levels of the knockout bacteria with three independent replicates. For the cell growth experiments, MRS medium was used, which included peptone 10 g/L, beef extract 10 g/L, yeast extract 5 g/L, C_6_H_5_O_7_(NH_4_)_3_ 2 g/L, Tween 80 1 ml/L, CH_3_COONa 5 g/L, K_2_HPO_4_ 2 g/L, MgSO_4_ 0.58 g/L, MnSO_4_ 0.25 g/L, pH adjusted to 6.2 with HCl solution and glucose 20 g/L. The pH level of the medium was measured by pH meter.

### Other statistical methods

Principle component analysis (PCA) was used to analyze the time and individual influence. Adonis analysis was conducted using the vegan package, and the permuted *P* value was obtained by 9999 permutations. Fisher Exact Test was used to obtain the enrichment of each functional cluster. Statistical figures and tables were obtained by a free statistical software R. Cluster was performed by the Cluster3.0 software and the heatmap was generated by Java TreeView (http://bonsai.hgc.jp/~mdehoon/software/cluster/software.htm).

### Reporting summary

Further information on research design is available in the Nature Research Reporting Summary linked to this article.

## Supplementary information

Supplementary Information

Supplementary Data 1

Reporting Summary

## Data Availability

The sequences reported in this paper have been deposited in the National Center for Biotechnology Information Sequence Read Archive under accession no. SRP065752.
