# Supplementary material for: Metagenomic and metatranscriptomic profiling of Lactobacillus casei Zhang in the human gut
Source: NPJ Biofilms Microbiomes. 2021 Jul 1;7:55. doi: 10.1038/s41522-021-00227-2 (PMC8249650; doi:10.1038/s41522-021-00227-2)
Supplement: Supplementary file 1 — Supplementary Information [file 41522_2021_227_MOESM1_ESM.pdf]

A

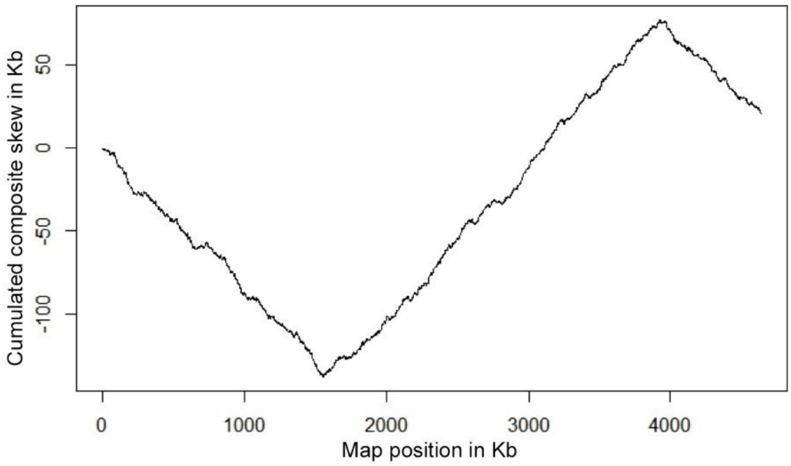

B

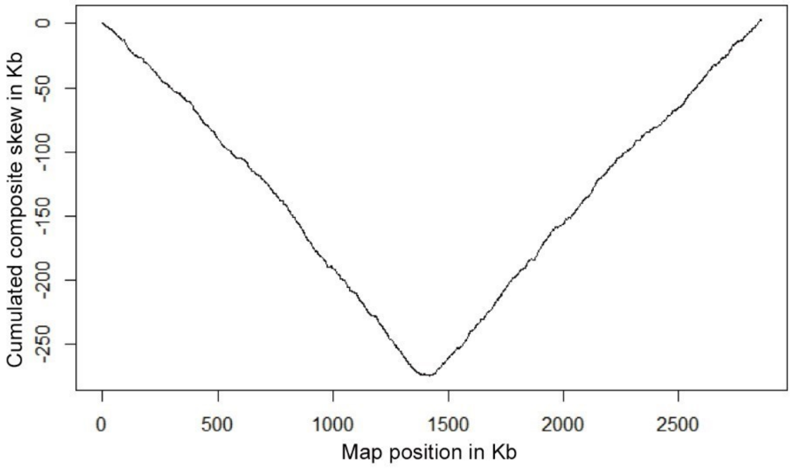

Supplementary Figure 1. Prediction of the replication origin by oriloc software.

(A) The predicted result of *Escherichia coli*, which was used as a positive control. The highest peak was the replication origin, and the lowest trough is the replication termination. (B) The predicted result of *L. casei* Zhang.

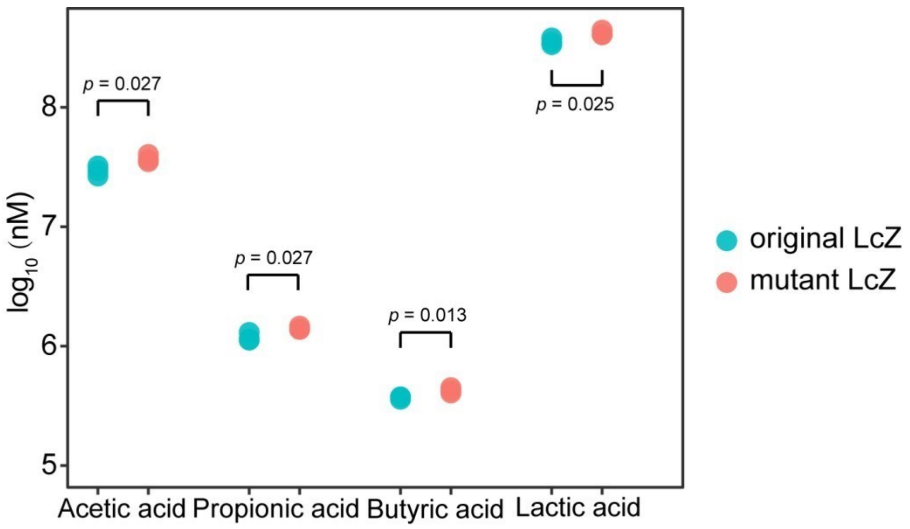

Supplementary Figure 2. Evaluation of the ability of original LcZ and mutant LcZ to produce SCFAs and lactic acid. We collected samples at 16h of cell growth, and three replicates were prepared (T-test, two-tailed).

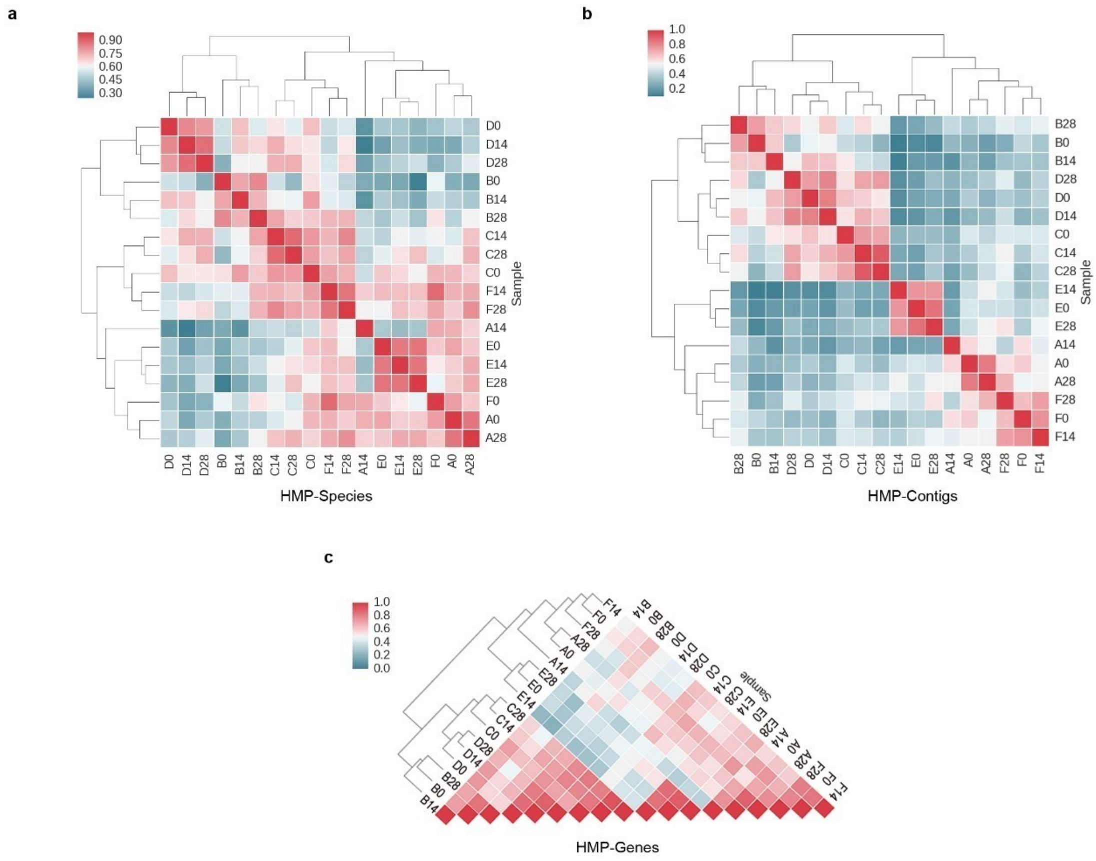

Supplementary Figure 3. Hierarchical clustering heatmap of sample correlation by calculating the Pearson correlation coefficient of the metagenomic abundance reflected by HMP mapping of all 18 samples. The maps for the Pearson correlation coefficient at the species (A), contigs (B) and gene (C) levels are shown.

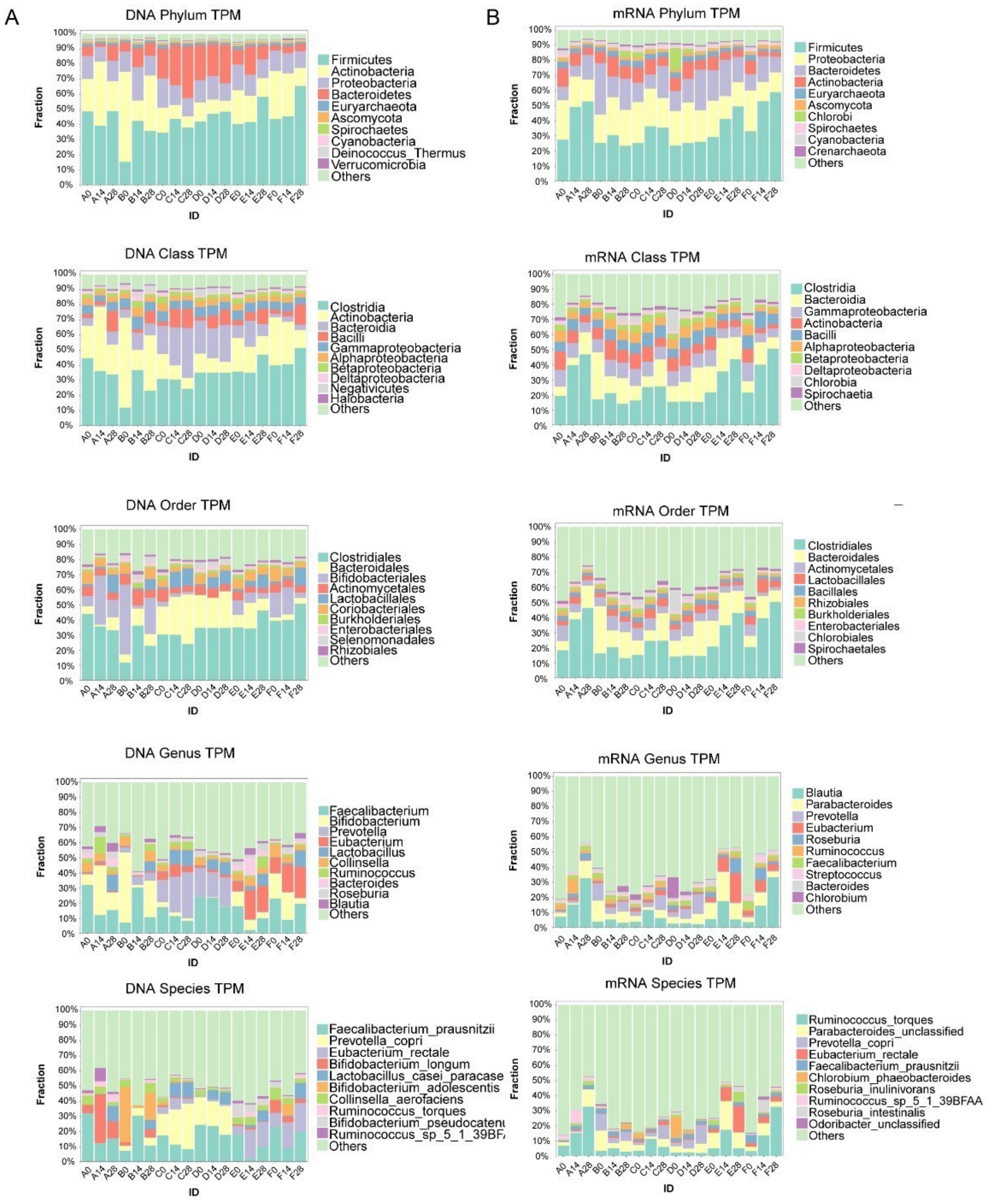

Supplementary Figure 4. Taxonomic summary of the observed relative abundance of taxa at different levels for metagenomics and metatranscriptomic reads by MetaPhlAn2 analysis. (**A**) Left panel represents the metagenomics taxa results. (**B**) Right panel represents for metatranscriptomic taxa results. From top to bottom, phylum, class, order, genus and species level are represented.

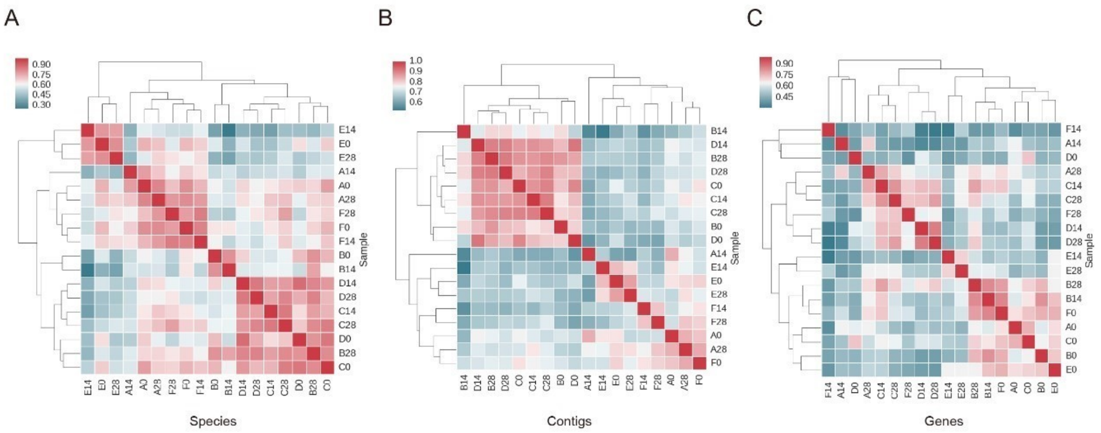

Supplementary Figure 5. Hierarchical clustering heatmap of sample classification by Pearson correlation coefficient of the metatranscriptomic abundance. The maps for the Pearson correlation coefficient at the species (A), contigs (B) and gene (C) levels are shown.

Supplementary Data Set containing Supplementary Table 1-7

Supplementary Table 1 23 Lactobacillus strains collected in HMP database

Supplementary Table 2 59 Lactobacillus species in MetaPhlAn2 database

Supplementary Table 3 Metagenomics and metatranscriptomics reads were mapped onto the genome sequence of L. casei Zhang

Supplementary Table 4 sRNA prediction result

Supplementary Table 5 sRNA expression level

Supplementary Table 6 Subject information collection of the six healthy young volunteers

Supplementary Table 7 Primer information for the validated genes in this study
