# Supplementary material for: Metagenomic and metatranscriptomic profiling of Lactobacillus casei Zhang in the human gut
Source: NPJ Biofilms Microbiomes. 2021 Jul 1;7:55. doi: 10.1038/s41522-021-00227-2 (PMC8249650; doi:10.1038/s41522-021-00227-2)
Supplement: Supplementary file 3 — Reporting Summary [file 41522_2021_227_MOESM3_ESM.pdf]

nature research

Corresponding author(s): Jiachao Zhang Zhihong Sun

Last updated by author(s): 2021-04-25

Reporting Summary

Nature Research wishes to improve the reproducibility of the work that we publish. This form provides structure for consistency and transparency in reporting. For further information on Nature Research policies, see our Editorial Policies and the Editorial Policy Checklist.

Statistics

For all statistical analyses, confirm that the following items are present in the figure legend, table legend, main text, or Methods section.

n/a	Confirmed	
☐	☒	The exact sample size (n) for each experimental group/condition, given as a discrete number and unit of measurement
☐	☒	A statement on whether measurements were taken from distinct samples or whether the same sample was measured repeatedly
☐	☒	The statistical test(s) used AND whether they are one- or two-sided<br>*Only common tests should be described solely by name; describe more complex techniques in the Methods section.*
☐	☒	A description of all covariates tested
☐	☒	A description of any assumptions or corrections, such as tests of normality and adjustment for multiple comparisons
☒	☐	A full description of the statistical parameters including central tendency (e.g. means) or other basic estimates (e.g. regression coefficient) AND variation (e.g. standard deviation) or associated estimates of uncertainty (e.g. confidence intervals)
☒	☐	For null hypothesis testing, the test statistic (e.g. F, t, r) with confidence intervals, effect sizes, degrees of freedom and P value noted<br>*Give P values as exact values whenever suitable.*
☒	☐	For Bayesian analysis, information on the choice of priors and Markov chain Monte Carlo settings
☒	☐	For hierarchical and complex designs, identification of the appropriate level for tests and full reporting of outcomes
☒	☐	Estimates of effect sizes (e.g. Cohen's d, Pearson's r), indicating how they were calculated

Our web collection on statistics for biologists contains articles on many of the points above.

Software and code

Policy information about availability of computer code

Data collection	The experiment was approved by the Ethics Committee of the Inner Mongolia Agricultural University (Hohhot, China). A written consent was obtained from every volunteer. Subjects were asked to orally intake 4 probiotic tablets consisting of a total of 10.6 Log10 CFU L. casei Zhang (LcZ) daily from Day 0 to 28. Stool samples were collected from the 6 subjects on Days 0, 14 and 28 in sterile containers and were kept refrigerated. Gut microbiota were sampled by non-invasively fecal collection. Stool samples were taken in duplicate by coring out feces with inverted sterile 1 mL pipette tips. These tips were then deposited in 15 mL Falcon tubes, and RNAlater was added to the tubes for meta-transcriptomic sequencing. Samples were collected in two minutes at home and stored temporarily at −20 . Samples were then transported on ice to the laboratory within 2 hours, and then stored in −80 freezers immediately.
Data analysis	Principle Component Analysis (PCA) was used to analyze the time and individual influence. Fisher Exact Test was used to obtain the enrichment of each functional cluster. Statistical figures and tables were obtained by a free statistical software R. Cluster was performed by the Cluster3.0 software and the heatmap was generated by Java TreeView (http://bonsai.hgc.jp/~mdehoon/software/cluster/software.htm).

For manuscripts utilizing custom algorithms or software that are central to the research but not yet described in published literature, software must be made available to editors and reviewers. We strongly encourage code deposition in a community repository (e.g. GitHub). See the Nature Research guidelines for submitting code & software for further information.

Data

Policy information about availability of data

All manuscripts must include a data availability statement. This statement should provide the following information, where applicable:

- Accession codes, unique identifiers, or web links for publicly available datasets
- A list of figures that have associated raw data
- A description of any restrictions on data availability

The sequences reported in this paper have been deposited in the National Center for Biotechnology Information Sequence Read Archive under accession no.

SRP065752.

Field-specific reporting

Please select the one below that is the best fit for your research. If you are not sure, read the appropriate sections before making your selection.

- [x] Life sciences
- [ ] Behavioural & social sciences
- [ ] Ecological, evolutionary & environmental sciences

For a reference copy of the document with all sections, see nature.com/documents/nr-reporting-summary-flat.pdf

Life sciences study design

All studies must disclose on these points even when the disclosure is negative.

Sample size	Stool samples were collected from the 6 subjects on Days 0, 14 and 28 in sterile containers and were kept refrigerated.
Data exclusions	Subjects who take antibiotics or other fermented products will be excluded.
Replication	Not applicable
Randomization	Not applicable
Blinding	Not applicable

Reporting for specific materials, systems and methods

We require information from authors about some types of materials, experimental systems and methods used in many studies. Here, indicate whether each material, system or method listed is relevant to your study. If you are not sure if a list item applies to your research, read the appropriate section before selecting a response.

Materials & experimental systems

n/a	Involved in the study	
☒	☐	Antibodies
☒	☐	Eukaryotic cell lines
☒	☐	Palaeontology and archaeology
☒	☐	Animals and other organisms
☐	☒	Human research participants
☒	☐	Clinical data
☒	☐	Dual use research of concern

Methods

n/a	Involved in the study	
☒	☐	ChIP-seq
☒	☐	Flow cytometry
☒	☐	MRI-based neuroimaging

Human research participants

Policy information about studies involving human research participants

Population characteristics	We collected metagenomic and meta-transcriptomic reads from the fecal samples taken from 6 healthy young volunteers (20 to 30 years old, three males and three females, labeled as A-E, Table S6) in an open-label clinical trial.
Recruitment	A written consent was obtained from every volunteer. Subjects were asked to orally intake 4 probiotic tablets consisting of a total of 10.6 Log10 CFU L. casei Zhang (LcZ) daily from Day 0 to 28. Stool samples were collected from the subjects on Days 0, 14 and 28 in sterile containers and were kept refrigerated. Gut microbiota were sampled by non-invasively fecal collection. Stool samples were taken in duplicate by coring out feces with inverted sterile 1 mL pipette tips. These tips were then deposited in 15 mL Falcon tubes, and RNAlater was added to the tubes for meta-transcriptomic sequencing. Samples were collected in two minutes at home and stored temporarily at −20 . Samples were then transported on ice to the laboratory within 2 hours, and then stored in −80 freezers immediately.
Ethics oversight	Ethics Committee of the Inner Mongolia Agricultural University (Hohhot, China)

Note that full information on the approval of the study protocol must also be provided in the manuscript.
